# Is Accessing of Words Affected by Affective Valence Only? A Discrete Emotion View on the Emotional Congruency Effect

**DOI:** 10.3389/fpsyg.2016.00916

**Published:** 2016-06-17

**Authors:** Xuqian Chen, Bo Liu, Shouwen Lin

**Affiliations:** Key Laboratory of Mental Health and Cognitive Science of Guangdong Province, Center for Studies of Psychological Application, School of Psychology, South China Normal UniversityGuangzhou, China

**Keywords:** affective valence, discrete emotion, prototypical emotion, congruency effect, embodied cognition

## Abstract

This paper advances the discussion on which emotion information affects word accessing. Emotion information, which is formed as a result of repeated experiences, is primary and necessary in learning and representing word meanings. Previous findings suggested that valence (i.e., positive or negative) denoted by words can be automatically activated and plays a role in many significant cognitive processes. However, there has been a lack of discussion about whether discrete emotion information (i.e., happiness, anger, sadness, and fear) is also involved in these processes. According to the hierarchy model, emotions are considered organized within an abstract-to-concrete hierarchy, in which emotion prototypes are organized following affective valence. By controlling different congruencies of emotion relations (i.e., matches or mismatches between valences and prototypes of emotion), the present study showed both an evaluative congruency effect (Experiment 1) and a discrete emotional congruency effect (Experiment 2). These findings indicate that not only affective valences but also discrete emotions can be activated under the present priming lexical decision task. However, the present findings also suggest that discrete emotions might be activated at the later priming stage as compared to valences. The present work provides evidence that information about discrete emotion could be involved in word processing. This might be a result of subjects’ embodied experiences.

## Introduction

Relatively enduring beliefs and predispositions toward specific objects or persons are called attitudes (Scherer, 2005). Automatic attitude activation is considered to be a mediating mechanism that plays a role in many significant cognitive processes (Fazio, 2001). In a series of priming studies (Klauer, 1997; Wentura, 1999; Hermans et al., 2001; Herring et al., 2013), significant emotion priming effects were demonstrated using prime-target pairs for which the emotional relation was manipulated, and the influence of affective valence, one of the components of attitudes consisting mostly of differential valence (Scherer, 2005), was most discussed. The evaluation associated with a prime appears to be suppressed when the target’s valence is incongruent with primes (Wentura, 1999). To date, researchers reasoned that presentation of an attitude object automatically activates from memory the valence that an individual associates with the object (Fazio, 2001).

Using the concept of affective valence, emotional priming effects were always discussed in a bipolar fashion: positive and negative. Namely, in most studies, a positive or negative prime stimulus (word or picture) was followed by a positive or negative target stimulus. Results showed that the reaction time was significantly shorter when prime and target shared the same valence (i.e., congruent condition: positive–positive or negative–negative) as compared to trials on which prime and target were of opposite valence (i.e., incongruent condition: positive–negative or negative–positive). Therefore, presentation of “cockroach” as the prime, the attitude toward which is evaluated negatively by an individual, appears to facilitate the activation of the negative evaluation of the target (e.g., disgusting, see Fazio, 2001).

Moreover, Kousta et al. (2009) found an abstractness effect in which words referring to positive or negative emotions are processed faster than neutral words, and words that have more emotional valence trigger a stronger residual latency advantage compared to those that do not, when variables such as imageability (a construct derived from dual coding theory) and rated context availability are held constant (Kousta et al., 2011). According to these findings, Kousta et al. (2011) proposed an embodied abstract semantics hypothesis, in which experiential information is considered as one of the two major types of information during word learning. The other type is language-based information (Vigliocco et al., 2009).

It seems that most of the above studies were arranged under the dimensional emotion framework. Beginning with Nowlis, most psychologists have described affect as a set of dimensions (see Russell, 1980 as review). In a valence-arousal circumplex model, these affective dimensions are considered to be interrelated in a highly systematic fashion. Interrelationships among affective concepts can be represented by a spatial model, in which valence (pleasantness or hedonic value) and arousal (bodily activation) are the two essential qualities (Russell, 1980; Izard, 1992; Reisenzein, 1994; Reisenzein and Spielhofer, 1994; Posner et al., 2005; Madrid and Patterson, 2014). However, the role of emotion information, one type of experiential information from our experience of our own inner states, is considered to be foundational (i.e., primary and necessary) in learning and representing meanings (Vigliocco et al., 2009). Words that denote emotional states, moods or feelings provide a crucial example of how a word may refer to an entity, even if it is not observable but resides within the organism, so that semantic representations can be developed. For example, a *nurse* is someone who offers help, and the word *nurse* should be more positive than words like *thief*, *killer*, or *robber*, etc. However, the *nurse* could be also a word denoting negative emotion, because a *nurse* offers injection and piles in our own experience. The fact that *a nurse offers injection and piles* could be learned as the crucial example of the word *nurse*. Then what kinds of embodied information about emotion can be acquired? In other words, is it appropriate to discuss emotional priming simply in a bipolar way? This is a question related to the issue of how people label, or make embodied statements about, their affective states.

Some researchers have claimed that this valence-arousal circumplex should be extended to a discrete framework, in which different numbers of basic/discrete emotion labels should be defined. That is, different from the modern dimensional theory (i.e., valence-arousal circumplex model), according to which feelings are described by the valence and arousal dimensional space, discrete emotion theory focuses on the original expressions describing clearly separable states (Scherer, 2005). According to this theory, most of the basic/discrete emotions play an important role in adapting to frequently occurring and prototypically patterned types of significant events in the life of organisms (Barrett, 1998; Scherer, 2005). For example, during emotional experience (i.e., “How do I feel?”) and emotion perception (e.g., “Is my baby afraid?” “Is my mother angry?” “Is my cat sad?”), both of which are related to the emotion knowledge that has been learned via prior experience, representations of internal sensations from the body and external sensations from the world become meaningful (Barrett, 2006; Niedenthal, 2007). Experience has also been argued to play an important role in developing concepts (Glenberg, 1997; Barsalou, 1999; Murphy, 2002; Zwaan, 2003; Barsalou et al., 2008), and the embodied simulation account is considered to be viable for understanding the processing and storing of emotional knowledge (Niedenthal et al., 2009). Therefore, the words “happiness,” “fear,” “sadness,” “anger,” “surprise,” and “interest” function as categorizing labels of emotion concepts that are related to the on-going activity that is realized in the brain (Barrett, 2006, 2009b; Barrett et al., 2009). Therefore, discrete labels, such as joy, anger, sadness and fear, were defined as prototypical emotions in the literature (Shaver et al., 1987; Scherer, 2005).

According to the above argument, one hypothesis can be made: all negatively evaluated emotions should not be the same, and the emotional priming effects should be more meticulously discussed. For example, both the feelings about “defeat” and “war” can activate the emotions of negative valence, but the former feeling should be sadness, whereas the latter one should be fear. As a consequence, it is plausible that both “sadness-war” and “fear-defeat” are emotionally congruent under the dimensional emotion view (positive–negative view), but incongruent under the discrete emotion view (“Sadness-defeat” and “fear-war” are emotionally congruent under both of the dimensional and the discrete emotion views.) Should the emotional priming effect be discussed under a different theoretical framework? We believed that the theoretical framework based only on the emotional valence dimension should be complemented with the more detailed analysis of discrete emotions when discussing emotional priming.

In the current study, emotional congruency effects were discussed under the discrete emotion view. That is, according to the discrete emotion view, though prime and target were both evaluated as words demoting negative emotions, they might be still considered to be incongruent emotions because they belong to different emotion categories. For example, in the current study one focus was anger and fear, which are both labels (or concepts) of negative valence. A key assumption was that when both are followed by a target (event word) denoting negative experience, there would be a significantly shorter reaction time when prime and target shared the same prototypical emotion (i.e., anger as a prime and a target word relating to experience of anger, or fear as a prime and a target word relating to experience of fear) as compared to trials on which prime and target are of different prototypical emotion (i.e., fear as a prime and target word relating to experience of anger, or anger as a prime and target word relating to experience of fear). However, this possibility has not yet been discussed. Therefore, the primary purpose of the present experiments was to provide evidence that besides emotional valence, discrete emotion also triggers congruent effects. To fulfill this goal, we arranged two priming lexical decision tasks (LDTs), in one of which (Experiment 1) emotional congruency was manipulated based on emotional valence, whereas in the other of which (Experiment 2) emotional congruency was manipulated based on prototypical emotions.

In addition, we also investigated whether it is the label or the experience/concept of discrete emotion that triggers the congruent effects. On the one hand, emotion labels serve to glue the various instances together into the single category of one emotion, even if they look very different from one another (Barrett, 2009b). In other words, emotion labels facilitate the learning of emotion categories. On the other hand, even trivial events (i.e., “seeing a cockroach”) can trigger distinct emotional feelings (Philippot et al., 2003), and the names of these events (i.e., “cockroach”) can then trigger the specific emotion (Fazio, 2001), because they might evoke memories of past experiences. However, discrete emotion information evoked by the name of events (i.e., “event words” in short) might not be as clear as those evoked by the label of the emotion. For investigating this statement, either the label of the emotion or event words were manipulated as the prime in the present study.

Though both valence and arousal are important qualities of affect (Barrett, 1998), dimensional labels and discrete labels are believed to be organized at different levels. Shaver et al. (1987) held that interrelated emotion categories are organized within an abstract-to-concrete hierarchy. Namely, the affective words are hierarchically organized first as reflecting their dimensional portrayal (i.e., positive and negative) and then as reflecting their discrete portrayal (e.g., prototypical emotions such as joy, anger, sadness and fear). Moreover, members of these prototypical emotions are stored at subordinate levels (e.g., amusement and pride are subordinate-level emotions of joy; Shaver et al., 1987).

Therefore, information from prototypical emotions might not be as strongly and quickly activated as affective valence. For testing this issue, stimulus onset asynchrony (SOAs; i.e., the interval between the onset of the prime and the onset of the target) was also manipulated in the present study. Researchers have paid great attention to evaluative priming effects under different SOAs, which is considered as a tool to provide insight to the time course of the activation processes underlying automatic affect/attitude activation. In a series of studies (Hermans et al., 1994, 2001; Hu, 2012), automatic evaluative processing has been evaluated under different SOAs (e.g., -150, 0, 50, 100, 150, 200, 300, 400, 1000 ms, etc.). It has been proposed that the process of automatic stimulus valence occurs at a very early stage in information processing, and researchers believe that the basic process is fast, efficient, and outside awareness (Hermans et al., 2001).

Using the sandwich masks technique, Hu (2012) reported a converse emotional congruent effect under short SOAs (i.e., 50, 100 ms), and the significant priming effect was reported under 150 ms. A comparison of short and long SOAs showed that affective priming effects were observed at an SOA of 300 ms, but not at a longer SOA of 1000 ms (Hermans et al., 1994; De Houwer et al., 1998); the conclusion was that a SOA of 300 ms is long enough to activate the associated evaluation so that responding to evaluative congruent targets was facilitated while responding to incongruent targets was inhibited (Hermans et al., 2001). According to the recent literature, especially a report using materials in Chinese (Hu, 2012), SOAs of 50, 150, 200, and 400 ms were selected.

Before the main study, the most important thing was to select the appropriate prototypical emotions categories and their corresponding event words. Some theories state that the ways people divide the emotional world into discrete categories are different across cultures, and even vary across individuals, and over time (Shaver et al., 1987; Barrett, 2006, 2009a,b). However, at least some researchers have stated that everyone within a culture shares roughly the same representation of emotion concepts (see Barrett, 2006 as review). Therefore, we selected prototypical emotions categories and their corresponding event words based on the culture of Chinese so that the influence from culture can be discreetly avoided. In the culture of Chinese, four essential prototypes (i.e., happiness, anger, sadness, and fear) are mainly discussed (Zhang, 2008). Some prototypical emotions in western culture (e.g., “perhaps” is considered as an emotion mentioned in Shaver et al., 1987 research) are not very distinguished in Chinese. In addition, in different cultures, numbers of positive and negative prototypical emotions are not equal (see samples provided in Barrett, 2006), and apparently, in Chinese there are no positive discrete emotions at the same intensity as negative discrete emotions such as sadness, anger, and fear, with the exception of happiness. More importantly, the influence of affective valence should be reduced when discussing effects of prototypical emotions. Four essential prototypes, happiness, anger, sadness and fear, were selected as materials, but only negative prototypes were used in Experiment 2.

Though we claimed that choosing only negative discrete emotions in Experiment 2 was subject to the goal of the present study, and also to the culture of China, there are still risks for making just conclusions because people might argue that the discrete emotion framework could be incomplete without discussing positive situations. In the literature, there were two hypotheses regarding different effects from positive and negative words: according to a negative-privileged view, negative valence is associated with a general slowdown of the processing of stimuli; but more recently, valence, regardless of polarity, was argued to equally facilitate processing due to the relevance of both negative and positive stimuli (see Kousta et al., 2009 as review). In addition, even according to the privileged hypothesis, negatively valenced stimuli invoke stronger behavioral responses than positive stimuli, even when arousal is held constant (Kousta et al., 2009). Though it was assumed that there should be discrete emotional congruency effects in the present study, congruency effects triggered by discrete emotional words might be not as significant as those triggered by evaluative emotional words. Selecting the more strongly activated emotional words (i.e., negative discrete words) should be appropriate for the present investigation.

Therefore, under the manipulations in the present study, the main hypotheses are as follow: (1) emotional congruency effects will be found in both valence priming (Experiment 1) and discrete emotional (Experiment 2) priming conditions; however, (2) this effect might occur later (i.e., in longer SOA conditions) under discrete emotional priming than under valence priming (i.e., in shorter SOA conditions) when SOAs were used as indexes, because the discrete emotional information would not be activated as strongly as valence information in the early stage of word processing.

## Pretest

The goal of this pretest was to select words denoting different emotions, which would be used in the following two experiments. In Experiment 1, affective words and event words denoting positive and negative emotions were needed, whereas in Experiment 2, the selected words denoted emotions that were negative but belonged to different prototypes of emotions. Therefore, in the present study, happiness was considered to be the positive prototype, while anger, sadness, and fear were considered to be the negative prototypes.

### Methods

#### Participants

Ninety university students from the South China Normal University (40 males, 50 females) participated in the experiment voluntarily, and were paid after the task.

#### Materials and Procedures

Four affective words, including happiness, sadness, anger, and fear, were selected as the cues, and printed in Chinese on A4 paper. Participants were instructed to freely associate and write down as many as words as possible that they associated with the cue. The part of speech (e.g., noun, adjective, verb, etc.) and relation between cues and word productions (e.g., synonymic, antonymic, etc.) were not restricted.

### Results and Analyses

Ninety questionnaires were delivered but an incomplete one was excluded in the final analyses. Results from the 89 available questionnaires showed 851 associated words were produced for *Happiness*, 833 for *Sadness*, 959 for *Anger*, and 828 for *Fear*. According to the relation between cues and productions, associate words were categorized into four groups: (1) synonyms, such as *happiness*-*delight*, (2) antonymic, such as *happiness*-*sorrow*, (3) life events, such as *sadness*-*failure*, (4) behavior caused by the emotion, such as *anger*-*vent*, (5) others which could not be categorized into the previous four groups, such as *anger*-*birds* (a name for a popular game). Numbers and frequencies of associated words are shown in **Table 1**, and a list of the most commonly associated words under each emotional cue is shown in **Table 2**.

**Table 1:** Descriptions on the frequencies of associated categories from the associated task (*N* = 89).

Affective words	Answer	Categories of associated words				
		Synonyms	Antonym	Life event	Relative behavior	Others
Happiness	Number	161	11	531	64	84
	Frequency (%)	18.92	1.29	62.40	7.52	9.87
Sadness	Number	152	2	535	82	62
	Frequency (%)	18.24	0.24	64.22	9.84	7.44
Anger	Number	89	1	558	193	118
	Frequency (%)	9.28	0.10	58.19	20.12	12.30
Fear	Number	148	2	550	40	88
	Frequency (%)	17.87	0.24	66.42	4.83	10.63

**Table 2:** Descriptions on the frequencies of first associated words in Experiment 1 (*N* = 89).

Affective words	Answer	Categories of associated words				
		Synonyms	Antonym	Life event	Relative behavior	Others
Happiness	Number	36	2	42	7	3
	Frequency (%)	40.45	2.24	47.19	7.87	3.37
Sadness	Number	21	0	47	17	4
	Frequency (%)	23.60	0	52.81	19.10	4.49
Anger	Number	25	1	45	10	8
	Frequency (%)	28.09	1.12	50.56	11.24	8.99
Fear	Number	29	0	52	6	1
	Frequency (%)	32.58	0	58.43	6.74	1.12

General chi-square test showed that associated frequency of life events was much higher than other associations, χ^2^= 53.71, *df* = 3, *p* < 0.001, η = 0.14. Further analyses showed that most of the words were associated with the experience of university life, including study, relationships, taking exams, love, family, health, competitions, seeking jobs, ability, and so on, which indicated that there is a strong mapping between affective and event words. More importantly, materials presenting event words could be chosen from associate words produced in the present pretest.

The top 16 associate event words and top 10 synonyms from each emotion group (i.e., happiness, sadness, anger, and fear) were selected. All these candidates were Chinese disyllable words. Because all selected words were relative to university students’ experience, but not all of them can be equally paired with word frequencies, familiarity ratings were used instead of word frequencies to control familiarity of materials in the present study. Thirty university students who did not participate in the main study gave five-point familiarity ratings (1 = very unfamiliar, 5 = very familiar) of all these life events and emotion primes. Words with an average rating higher than 3.5 were kept as the final materials (see Appendices A–C).

## Experiment 1

The goals of Experiment 1 were (1) to replicate the emotional congruent effect in which reaction time was significantly shorter when the prime and target shared the same valence as compared to trials on which prime and target were of opposite valence under four SOAs levels (i.e., 50, 150, 200, 400 ms); and (2) to compare such a congruent effect under the affective word priming condition and event word priming condition.

### Methods

#### Participants

One hundred students (27 males, 73 females, ages from 18 to 23, *M* = 21.08) from South China Normal University participated in the present study. They were volunteers and were paid after participation.

#### Design

Stimuli in a priming LDT were manipulated in a 2^∗^2^∗^4 repeated-measures factorial design with evaluative congruency (congruent vs. incongruent) and priming type (affective word vs. event word) as within group factors, SOAs (50/150/200/400 ms) as a between group factor, and latencies and error rates as the dependent variables. Priming (affective word or event word) and probes (event word) sharing the same affective valence were considered as the congruent condition, whereas those representing different affective valence were considered as the incongruent condition. All participants were divided into four groups randomly, 25 students for each group, and fulfilled only one SOA condition.

#### Materials

Materials were received from the pretest in which 16 affective words and 32 event words were selected. Affective words, half of which were positive whereas half of which were negative, were all used as primes. Event words, 16 of which denoted positive emotion 16 of which denoted negative emotion, were used as primes or targets. Therefore, there were eight positive event primes, eight negative event primes, eight positive event targets, and eight negative event targets. In addition, another 16 pseudo words were created as the filler probes so that the number of correct *Yes* and *No* responses could be balanced. To establish congruencies in affective valence between primes and probes (affective-event or event-event), four types of prime-probe word pairs were manipulated: (1) affective-event word pairs with congruent evaluation (e.g., positive: 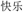
happiness-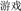 play games; negative: 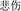 sadness-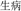 sick), (2) affective-event word pairs with incongruent evaluation (e.g., 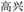 joyfulness-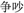 quarrel; 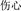 feeling bad-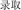 being accepted), (3) event-event word pairs with congruent evaluation (e.g., positive: 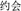 dating-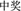
prizewinning; negative: 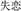 love fails-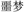 nightmare), (4) event-event word pairs with incongruent evaluation (e.g., 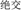 break of relations-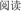 reading; 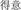 pleased-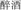 drunk).

#### Procedure

A priming LDT without feedback was used. The experimental software E-Prime presented the stimuli and recorded reaction times. Control files were constructed to display on the 17 inch IBM (9512-AB1) monitor (Screen resolution: 1024 × 768 Pixels), and participants were tested individually in a sound-proof room. Response errors and reaction times, measured from the onset of words, were automatically recorded by computers for each participant and each item. A trial consisted of the following sequence of events: a fixation point (+) was presented for 500 ms in the center of the screen, then right after the fixation cross disappeared a priming word was presented for 50, 150, 200, or 400 ms (different SOAs). Probes were presented at the center of the computer screen right after the offset of the priming and remained on the screen for maximum 3000 ms.

Participants were asked to judge as quickly and as accurately as possible whether or not the probe in each trial was a real word, and to press the corresponding button on a response box. The inter-trial interval was 500 ms (blank screen) and then the next trial started. If the participant failed to respond in 3000 ms, the trial was terminated and the trial was recorded as an error. Participants received 10 practice trials, for which feedback was provided. No feedback was provided during the experiment.

### Results

Participants’ mean latencies (based on untrimmed correct responses) and error rates are summarized in **Table 3**.

**Table 3:** Means of latencies (ms) and error rates (%) in Experiment 1 (based on evaluative congruency).

		SOA							
Evaluative congruency		Affective word priming				Event word priming			
		50ms	150 ms	200 ms	400 ms	50ms	150 ms	200 ms	400 ms
Latencies	Congruent	658 ± 94	652 ± 70	728 ± 107	705 ± 92	595 ± 69	613 ± 82	638 ± 97	618 ± 58
	Incongruent	684 ± 114	670 ± 93	809 ± 128	797 ± 124	630 ± 99	652 ± 61	696 ± 106	669 ± 80
Error rates	Congruent	6.2 ± 7.7	4.0 ± 5.5	1.8 ± 2.8	5.8 ± 10.5	6.2 ± 8.5	4.2 ± 4.5	2.6 ± 4.4	6.0 ± 7.2
	Incongruent	8.8 ± 9.2	6.4 ± 6.4	2.2 ± 3.3	6.3 ± 9.6	5.8 ± 7.3	4.8 ± 3.3	2.2 ± 2.9	7.0 ± 10.7

Results of the analyses on latency showed a significant difference between congruent and incongruent priming, *F*(1,96) = 24.60, *MSE* = 250299, *p* < 0.001, η^2^ = 0.20, and between affective word priming and event word priming, *F*(1,96) = 52.89, *MSE* = 544573, *p* < 0.001, η^2^= 0.36. In addition, a main effect of SOAs was found, *F*(3,96) = 10.08, *MSE* = 140450, *p* < 0.001, η^2^= 0.24. Further LSD tests showed that shorter latencies were caused by shorter SOAs (50, 150 ms), rather than by longer ones (200, 400 ms). However, differences between 50 and 150 ms priming, and between 200 and 400 ms priming, were not found. The interaction between priming type and congruency did not reach significance, *F* < 1, ns. In addition, the interaction between priming type and SOAs, and the three-way interaction, were missing, *F*s < 1, ns.

Results of the analyses on error rates showed that fewer error rates were produced under the 200 ms priming condition than the other three SOA conditions, *F*(3,96) = 9.67, *MSE* = 0.046, *p* < 0.001, η^2^= 0.24, but a difference among the latter three SOA conditions was not found. However, a main effect of emotional congruency, *F*(1,96) = 1.22, *MSE* = 0.008, *p* > 0.10, and priming types, *F*(1,96) < 1, *ns*, were both missing. In addition, the interactions did not reach significance, *F*’s < 1, ns.

### Discussion

Experiment 1 was mainly focused on whether in a priming LDT event words can elicit a similar emotion priming effect as compared to affective words. As expected, there was a facilitation in priming LDT when the priming and targets were of congruent emotional valence (i.e., positive–positive, or negative–negative), whereas such priming effect was missing when priming and targets were of incongruent emotional valence (i.e., positive–negative, or negative–positive), whether affective or event words were used for priming. These findings are important because they replicate earlier works on the emotional congruency effect (Wentura, 1999; Fazio, 2001). More importantly, Experiment 2 builds on these results in that by controlling congruencies in a discrete emotion level, the discrete emotion view on emotional effect can be tested.

## Experiment 2

The goal of Experiment 2 was to test the congruent effect with discrete emotions as primes to elicit the emotional congruency effect. Namely, in contrast to the traditional positive–negative categorization, emotions were divided into discrete categories, such as happiness, sadness, anger, and fear. The main hypothesis was that participants would take longer to respond in the priming LDT when prime and target were of different discrete emotion categories (e.g., sadness and fear), although, they were of the same valence (e.g., negative). To avoid an evaluative congruent effect, only one valence (negative) was chosen in the present experiment. That is, of the four basic emotions, only happiness is positive whereas the other three are negative, so materials belonging to the sadness, anger, and fear categories were selected.

### Methods

#### Participants

Participants in Experiment 1 also took part in Experiment 2.

#### Design

The design was identical to that of Experiment 1 except that emotional congruency was defined based on the discrete emotion. Namely, priming (affective word or event word) and probes (event word) sharing the same discrete emotion (i.e., sadness, fear, anger) were considered as the congruent emotion category group, whereas, those representing different discrete emotions were considered as the incongruent emotion category group.

#### Materials

Materials were generated in the pretest. To avoid the effects of positive and negative evaluation, only materials related to sadness, anger, and fear (but not happiness) were used. There were 24 affective words and 48 event words. Affective words, eight of which denoted anger, eight of which denoted sadness, and the rest of which denoted fear, were all used as primes. Event words, 16 of which denoted anger 16 of which denoted sadness, and the rest of which denoted fear, were used as primes or targets. Therefore, there were eight angry-event primes, eight sad-event primes, eight fear-event primes, eight angry-event targets, eight sad-event targets, and eight fear-event targets. To establish congruencies in emotion category between primes and probes (affective-event or event-event), four types of prime-probe word pairs were manipulated: (1) affective-event word pairs with congruent emotion (e.g., sadness: 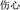 sadness-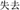 lose; fear: 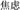 anxious-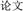 writing thesis; anger: 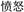 rage-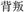 betray), (2) affective-event word pairs with incongruent emotion (e.g., 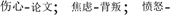

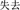
), (3) event-event word pairs with congruent emotion (e.g., sadness: 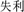 defeat-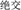 break off relations; fear: 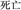 death-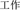 working; anger: 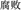 corruption-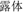 naked), (4) event-event word pairs with incongruent emotion (e.g., 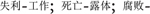

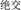
).

#### Procedure

The procedure was identical to that in Experiment 1.

### Results

#### General Analysis

Participants’ mean latencies (based on untrimmed correct responses) and error rates are summarized in **Table 4**.

**Table 4:** Means of latencies (ms) and error rates (%) in Experiment 2 (based on discrete emotional congruency).

Discrete emotional congruency	SOA							
	Affective word priming				Event word priming			
		50ms	150 ms	200 ms	400 ms	50ms	150 ms	200 ms	400 ms
Latencies	Congruent	616 ± 60	641 ± 75	708 ± 97	696 ± 89	627 ± 61	631 ± 87	658 ± 90	615 ± 51
	Incongruent	694 ± 102	672 ± 104	751 ± 106	828 ± 123	615 ± 97	640 ± 62	653 ± 88	690 ± 113
Error rates	Congruent	5.6 ± 5.6	2.0 ± 3.8	1.8 ± 2.4	6.0 ± 10.8	6.6 ± 8.3	5.8 ± 7.5	3.0 ± 4.1	5.4 ± 8.0
	Incongruent	10.08 ± 7.4	7.0 ± 6.0	1.2 ± 2.2	8.8 ± 13.8	5.6 ± 7.3	5.6 ± 4.8	2.6 ± 3.3	6.5 ± 9.2

Results of the analyses on latency showed a significant difference between emotional congruent and incongruent priming, *F*(1,96) = 17.10, *MSE* = 194323, *p* < 0.001, η^2^= 0.15, and between affective word priming and event word priming, *F*(1,96) = 33.35, *MSE* = 360408, *p* < 0.001, η^2^= 0.26. In addition, a main effect of SOAs was found, *F*(3,96) = 7.22, *MSE* = 116134, *p* < 0.001, η^2^= 0.18. Further LSD tests showed that shorter latencies were caused by shorter SOAs (50, 150 ms), rather than by longer ones (200, 400 ms). However, differences between 50 and 150 ms priming, and between 200 and 400 ms priming, were not found. The interaction between priming type and congruency reached significance, *F*(1,96) = 5.94, *MSE* = 72763, *p* < 0.05, η^2^= 0.06. Further simple effect analyses were used to test specific comparisons that might explain this interaction. These analyses showed that the congruency effect was generally significant under the affective word priming condition (*p* < 0.001), but not under the event word priming condition (*p* > 0.10). In addition, the interaction between priming type and SOAs was significant, *F*(3,96) = 3.68, *MSE* = 39788, *p* < 0.05, η^2^= 0.10, and the interaction between congruency and SOAs reached significance, as well, *F*(3,96) = 3.60, *MSE* = 40943, *p* < 0.05, η^2^= 0.10. Simple effects analyses on the priming type^∗^SOAs interaction showed significant advantages on emotional words priming occurred only on SOA = 200 ms (*p* < 0.05), and SOA = 400 ms (*p* < 0.05); while simple effect analyses on the congruency^∗^ SOAs interaction indicated that significant emotional congruency effects occurred when SOA = 400 ms (*p* < 0.05) rather than other three SOA conditions (*p*s > 0.05). However, the three-way interaction was missing, *F* < 1, ns.

Results of the analyses on error rates showed that fewer errors were produced under the 200 ms priming condition than the other three SOA conditions, *F*(3,96) = 9.43, *MSE* = 0.043, *p* < 0.001, η^2^= 0.23, but no difference among the latter three SOA conditions was found. However, a main effect of emotional congruency, *F*(1,96) = 0.82, *MSE* = 0.005, *p* > 0.10, and of priming types, *F*(1,96) = 0.35, *MSE* = 0.002, *p* > 0.10, were both missing. In addition, no interaction reached significance [priming type ^∗^ SOAs: *F*(3,96) = 1.18, *MSE* = 0.006, *p* > 0.10, emotional congruency ^∗^ SOAs: *F*(3,96) = 0.64, *MSE* = 0.004, *p* > 0.10, priming type ^∗^ emotional congruency: *F*(1,96) = 2.21, *MSE* = 0.013, *p* > 0.05, emotional congruency ^∗^ priming type ^∗^ SOAs: *F*(3,96) = 0.60, *MSE* = 0.003, *p* > 0.10].

#### Further Analysis of Congruency Effect Under Event Word Priming Across Experiments

In the present study, the main focus was emotional congruency effects in event word priming conditions. Therefore, latency data from both experiments under event word priming were also submitted to 2 (Experiments)^∗^2 (Priming types) ^∗^4 (SOAs) repeated-measures ANOVAs. Results showed a significant difference between congruent and incongruent priming, *F*(1,96) = 11.32, *MSE* = 98538, *p* < 0.01, η^2^= 0.11, and among SOAs, *F*(3,96) = 2.60, *MSE* = 36516, *p* = 0.05, η^2^= 0.08. However, the difference between experiments did not reach significance, *F* < 1, ns. In addition, the interaction between priming type and experiments reached significance, *F*(1,96) = 5.72, *MSE* = 20401, *p* < 0.05, η^2^= 0.06, and the three-way interaction was marginally significant, *F*(3,96) = 2.53, *MSE* = 9013, *p* = 0.06, η^2^= 0.07, whereas other interactions were missing [Experiment^∗^SOAs: *F* < 1, ns; Congruency^∗^SOAs: *F*(3,96) = 1.41, *MSE* = 12277, *p* > 0.10].

Based on the three-way interaction, further simple effect analyses were conducted to test differences between congruent and incongruent event word priming under different SOA conditions separately (details are in **Table 5**). Different from emotional congruency effects occurring at the late SOA stage (SOA = 400 ms) under discrete emotional priming (Experiment 2), a significant priming effect occurred at the early SOA stage (SOA = 50 ms) when congruency was defined in terms of positive–negative emotion (Experiment 1).

**Table 5:** *T*-test between congruent and incongruent event priming under different SOAs (*df* = 24).

	SOA = 50 ms		SOA = 150 ms		SOA = 200 ms		SOA = 400 ms	
	*t*	*p*	*t*	*p*	*t*	*p*	*t*	*p*
EXP1 EXP2	2.312 0.557	0.03^∗^ 0.58	1.894 0.556	0.07 0.58	2.082 0.177	0.048^∗^ 0.86	2.404 3.185	0.024^∗^ 0.004^∗∗^

*^∗∗∗^p < 0.001, ^∗∗^p < 0.01, ^∗^p < 0.05.*

### Discussion

The novelty of Experiment 2 is that congruency of discrete emotions (i.e., sadness, anger, and fear), all of which were of negative valence, appeared to affect performance in the LDT. However, different from the results of Experiment 1, in which more dimensional emotion information (i.e., positive vs. negative) was manipulated, the congruency effect in Experiment 2 occurred only under the SOA level of 400 ms and when primes were event words. One of the plausible reasons was that affective information from emotional labels could be more easily activated than that from event words. However, another more possible reason was that life events were often displayed with discrete emotions in everyday expressions. This means that at least a part of the present priming effect under the emotion priming condition, which was larger and more stable than the effect elicited by event priming, was affected by the highly frequent collocation. Therefore, the priming effect under the event word priming condition should be more pronounced than under the emotion priming condition. The present results indicate that the experiment triggered a discrete emotional congruent effect at a later stage (i.e., SOA = 400 ms) as compared to that triggered by general emotional valence (SOA = 50 ms in Experiment 1). This can be considered as evidence that discrete emotion information was activated during the accessing of event words, but this information was much weaker and activated later than in response to words denoted by positive–negative valence.

## General Discussion

The present study tested whether discrete emotion information can be activated during word processing. In the literature, researchers the emotional dimension was mostly characterized as positive or negative, the emotional congruency effect has yet to be discussed at the discrete emotion level. In addition, we focused on whether discrete emotion can elicit congruency effects as early as the evaluation of emotion. Robust evaluative congruency effects at the SOA levels of 50, 200, and 400 ms (Experiment 1), and the discrete emotional congruency effect at the SOA level of 400 ms (Experiment 2) under the event word priming condition, indicated that not only general affective valence but also discrete emotions could be activated under the present priming LDT. However, discrete emotions were activated at the later priming stage as compared to general emotional valences. These findings are important because they replicate earlier work on the one hand, but also extend that work by moving from general to discrete emotion. Moreover, the results provide evidence for the hierarchy model of emotion.

It is frequently assumed that people spontaneously evaluate incoming stimuli in terms of bipolar valence, such as pleasant or unpleasant, liked or disliked, good or bad (Klauer, 1997). Altarriba et al. (1999) and Altarriba and Bauer (2004) noted that valence of affective words affects word recognition and retrieval (Altarriba and Bauer, 2004). This effect can be explained by the semantic activation model (Altarriba and Bauer, 2004). However, Kousta et al. (2011) claimed that these findings are consistent with the embodied theory. Research by Kousta et al. (2011) showed that abstract words with affective associations are acquired earlier than are neutral abstract words. Therefore, contrary to the earlier idea that mappings between word and world occur during concrete word learning, Vigliocco et al. (2009) posited a more general explanation. Namely, emotional states, moods or feelings denoted by words represent a mapping from the word to the world, and affect word accessing, no matter which kind (i.e., concrete or abstract) of word it is.

Although, the embodied approaches have been extended to the representation and processing of the valence of experiences of word meanings, it has been far less discussed how a discrete embodied experience could be valid in word accessing. During emotional development, emotions should not be learned only dimensionally as positive, negative, and neutral, but also discretely as happiness, anger, sadness or fear (Shaver et al., 1987). That is, there should be links between particular appraisals and particular emotions (Roseman et al., 1990). As a consequence, it becomes possible that various systematic attempts can be triggered by different emotions to regain control by shifting attention to the threat and physiological arousal in preparation for behavioral responses (Ekman, 2003; Rivers et al., 2007). Therefore, preparation for behavioral responses to “defeat” and “war” are different because experiences of “sadness” and “fear” are different. However, there has been a lack of discussion about whether emotion information or emotional experience affects our word learning and accessing at the discrete emotion level.

Results of Experiment 1 provided evidence that different kinds of words elicited similar emotional congruency effects under the affective valence condition. Based on these results, which can be seen as supporting the argument by Vigliocco et al. (2009), we further investigated a similar effect under the superordinate level in Experiment 2. Results of Experiment 2 indicated that discrete emotion also affects word processing. Different from most of the recent research in which life events were always categorized as positive and negative (Robinson and Kirkeby, 2005; Robinson and Hippel, 2006), Experiment 2 categorized words referring to different discrete emotions belong to the three negative prototypes (namely whether words have sad, angry, or anxious connotations). We found an advantage for the congruent condition in comparison to the incongruent condition, in which congruent emotion facilitation was found when primes and probes belonged to the same prototypical emotion when all materials were of negative valence. In other words, specific prototypical emotion information was accessed in the present priming LDT.

Moreover, the priming effect was found to be robust and stable under affective word priming, no matter whether the emotions were dimensionally or discretely manipulated. This finding might be the evidence that emotion information was more easily activated by clear emotion labels. However, it was not completely clear that such a priming effect elicited by affective words was completely a congruency effect. Affective-event word pairs were generated by a word-association test. As a consequence, the priming effects might have been partially triggered by the highly frequent collocation between affective words and events in everyday expressions. Discrete affective words and event words in the congruent condition are frequently used in everyday expressions (e.g., *I am angry at his defection*, or *I am happy that you passed the exam*). A specific emotional label can semantically activate several candidate events, which can be regarded as preparation for the upcoming LDT, and responses for the congruent trails will be quickened as compared to the incongruent trails. Should the present findings be more consistent with the language-based view (Vigliocco et al., 2009), rather than embodied emotion view?

Therefore, results of event word priming, in which the influence of highly frequent collocation was excluded, are more convincing for the present discussion. According to the SOA effect, under event word priming, an emotional congruency effect was found at the early stage when congruency of affective valence was manipulated (i.e., positive–negative, in Experiment 1), but at the late stage when congruency of discrete emotion was manipuated (i.e., sadness, anger, and fear, in Experiment 2). Apparently, activation of the discrete emotion information was not as fast as that of affective valence.

We believe that the present results are consistent with the hierarchical view of emotion, which is compatible with the bipolar view of the emotional congruency effect. Shaver et al. (1987) proposed a dynamic model of the emotion process implicit in emotion episodes. According to this model, emotions are conceptualized as beginning with an interpretation of events as positive or negative; then, one of the basic emotions, namely one of the prototypical emotions in the present study, is elicited, accompanied by characteristic action tendencies, cognitive biases, and physiological patterns that also arise automatically. This emotion-related information is processed every day, and people can comprehend an event in either a more detailed or more general way. For example, a detailed description such as “a sad woman” is more cognitively vivid and imaginable than a description such as “a woman who is experiencing negative emotion” (Shaver et al., 1987). Therefore, existence of a discrete emotional congruency effect is acceptable.

In addition, according to this dynamic model, experience of emotion could be described under different dimensions: valence (i.e., positive or negative), potency (i.e., weak or strong), and activity (i.e., low or high), all of which are forms of experiential information that would affect word processing. Therefore, experiential information of a word can be processed evaluatively (i.e., positive or negative; Fazio, 2001), and the stronger the feeling is, the faster the process is (i.e., positive/negative vs. neutral; Kousta et al., 2009). More importantly, as mentioned in the three-dimensional solution model (Shaver et al., 1987), anger-related emotions tend to be high in potency, sadness-related emotions tend to be low in potency, and fear tends to be intermediate in potency. In other words, results of the present study indicated that the potency can also affect word processing. We believed that this feeling of potency comes from subjects’ everyday experience.

## Conclusion

This study provides evidence that emotion information denoted by a word can be considered a prominent feature that affects word accessing, and the results are discussed in terms of the extent to which discrete emotion information is involved in this process. Emotion information as a prominent feature appears beneficial on several levels. Firstly, emotion information is primary and necessary in learning and representing word meanings (Vigliocco et al., 2009), and as a result of repeated experiences, emotions are organized within an abstract-to-concrete hierarchy (Shaver et al., 1987). Therefore, both the bipolar feature (i.e., positive-to-negative valences) and the subordinate feature (i.e., prototypical emotions) affect word accessing. Secondly, activation of emotion information varies under different levels. Valence of emotion denoted by words is fast and strongly activated, and then followed by activation of subordinate emotion information. Thirdly, affective words elicit larger priming effects than event words do. This priming effect might be partially triggered by highly frequent collocation between primes and probes in everyday expressions, which was not tested in the present study.

Taken together, the findings suggest that discrete emotion information produces congruency effects similar to those triggered by general affective valence. In addition, consistent with the hierarchical model of emotion, in which emotion information is organized within an abstract-to-concrete hierarchy, these discrete emotion effects, as compared to dimensional ones, occur at the later stage of word processing.

## Author Contributions

XC: idea, design, management, writing; BL: Experiments 1 and 2; SL: pretest.

## Conflict of Interest Statement

The authors declare that the research was conducted in the absence of any commercial or financial relationships that could be construed as a potential conflict of interest.
