# Supplementary material for: Is Accessing of Words Affected by Affective Valence Only? A Discrete Emotion View on the Emotional Congruency Effect
Source: Front Psychol. 2016 Jun 17;7:916. doi: 10.3389/fpsyg.2016.00916 (PMC4911411; doi:10.3389/fpsyg.2016.00916)
Supplement: Supplementary file 1 [file Table_1.DOCX]

Appendix A: Materials used in Experiment 1.

| Primes | | | | Probes | |
| --- | --- | --- | --- | --- | --- |
| Emotion words | | Life Event words | | Life Event words | |
| Positive | Negative | Positive | Negative | Positive | Negative |
| 快乐  高兴  喜悦  幸福  快活  愉悦  得意  开心 | 悲哀  难过  沮丧  悲伤  心酸  哀愁  伤心  伤感 | 旅游  (travelling)  放假  (holiday)  运动  (having sports)  约会  (dating)  上网  (surf on-line)  逛街  (shopping)  回家  (go home)  卧谈  (bedtime chatting) | 离别  (bid farewell)  挂科  (flunk)  开会  (having meeting)  失恋  (love fails)  下雨  (raining)  熬夜  (stay up late)  绝交  (break off relations)  争吵  (quarrel) | 睡觉  (enough sleeping)  游戏  (play games)  网购  (online shopping)  宵夜  (bedtime snacks)  中奖  (prizewinning)  升职  (promotion)  录取  (being accepted)  阅读  (reading) | 面试  (job interview)  噩梦  (nightmare)  打针  (have an injection)  生病  (sick)  独处  (stay alone)  呕吐  (vomit)  停电  (electricity fails)  醉酒  (drunk) |
