# Supplementary material for: Is Accessing of Words Affected by Affective Valence Only? A Discrete Emotion View on the Emotional Congruency Effect
Source: Front Psychol. 2016 Jun 17;7:916. doi: 10.3389/fpsyg.2016.00916 (PMC4911411; doi:10.3389/fpsyg.2016.00916)
Supplement: Supplementary file 2 [file Table_2.DOCX]

Appendix B: Emotion words and life events as primes used in Experiment 2

| Emotion words | | |  | Life-event words | | |
| --- | --- | --- | --- | --- | --- | --- |
| Sadness | Anger | Fear |  | Sadness | Anger | Fear |
| 悲哀  难过  沮丧  悲伤  心酸  哀愁  伤心  伤感 | 愤怒  恼怒  发火  生气  气愤  怨愤  盛怒  震怒 | 忧虑  焦虑  着急  操心  担忧  紧张  顾忌  苦恼 |  | 吵架 (quarrel)  孤独 (be alone)  变丑 (disfigure)  肥胖 (fat)  离婚 (divorce)  误解 (misunderstand)  遗忘 (forget)  失利 (defeat) | 冤枉 (be treated unjustly)  嘲笑 (be laughed at)  腐败 (corruption)  谎言 (lies)  插队 (cut in line)  爽约 (be stood up)  打搅 (be disturbed)  隐瞒 (conceal) | 死亡 (death)  地震 (earthquake)  疾病 (ill)  老鼠 (rat)  前途 (future)  灵魂 (ghost)  战争 (war)  补考 (resit) |
