# Supplementary material for: Is Accessing of Words Affected by Affective Valence Only? A Discrete Emotion View on the Emotional Congruency Effect
Source: Front Psychol. 2016 Jun 17;7:916. doi: 10.3389/fpsyg.2016.00916 (PMC4911411; doi:10.3389/fpsyg.2016.00916)
Supplement: Supplementary file 3 [file Table_3.DOCX]

Appendix C: Life events as targets used in Experiment 2

|  | Sadness | Anger | Fear |
| --- | --- | --- | --- |
| 1  2  3  4  5  6  7  8 | 离别 (bid farewell)  挂科 (flunk)  开会 (having meeting)  失恋 (love fails)  考试 (having exam)  下雨 (raining)  绝交 (break off relations)  失去 (lose) | 背叛 (betray)  失窃 (be stolen)  欺骗 (cheat)  露体 (naked)  插队 (cut in line)  停电 (power failure)  爽约 (being stood up)  等车 (wait for a bus) | 工作 (working)  失眠 (agrypnia)  毕业 (graduate)  论文 (writing thesis)  开学 (new semester)  军训 (military training)  借钱 (borrow money)  评优 (evaluate) |
